# Quantifying Preferences of Farmers and Veterinarians for National Animal Health Programs: The Example of Bovine Mastitis and Antimicrobial Usage in Switzerland

**DOI:** 10.3389/fvets.2017.00082

**Published:** 2017-06-02

**Authors:** Bart H. P. van den Borne, Felix J. S. van Soest, Martin Reist, Henk Hogeveen

**Affiliations:** ^1^Vetsuisse Faculty, Veterinary Public Health Institute, University of Bern, Liebefeld, Switzerland; ^2^Business Economics Group, Wageningen University & Research, Wageningen, Netherlands; ^3^Federal Food Safety and Veterinary Office, Liebefeld, Switzerland; ^4^Department of Farm Animal Health, Utrecht University, Utrecht, Netherlands

**Keywords:** mastitis, dairy cows, adaptive choice-based conjoint analysis, multivariate multiple regression, animal disease program

## Abstract

Bovine udder health in Switzerland is of a relatively high level. However, antimicrobial usage (AMU) seems high in comparison to other European countries also. A new udder health and AMU improvement program could improve this situation but it is uncertain whether there is support from the field. This study aimed to quantify preferences of dairy farmers and veterinarians for the start and design characteristics of a new national udder health and AMU improvement program in Switzerland. A total of 478 dairy farmers and 98 veterinarians completed an online questionnaire. Questions on their demographics and their mindset toward AMU were complemented with an adaptive choice-based conjoint interview, a novel conjoint analysis technique to quantify preferences of respondents for characteristics of a product for which multiple trade-off decisions must be made (here a bovine udder health and AMU improvement program). The conjoint analysis was followed by a multivariate multiple regression analysis to identify groups of respondents with different program design preferences. Logistic regression models were used to associate covariates with respondents’ preference to start a new udder health and AMU improvement program. Most farmers (55%) and veterinarians (62%) were in favor of starting a new voluntary udder health and AMU improvement program, but the program design preferences agreed moderately between the two stakeholder groups. Farmers preferred an udder health and AMU improvement program that did not contain a penalty system for high AMU, was voluntary for all dairy herds, and aimed to simultaneously improve udder health and reduce AMU. Veterinarians preferred a program that had the veterinary organization and the government taking the lead in program design decision making, did not contain a penalty system for high AMU, and aimed to simultaneously improve udder health and reduce AMU. Differences between groups of farmers and veterinarians concerning their start preference were identified. Also, the magnitude of various program design preferences changed for farmers with different opinions toward AMU. The information obtained from this study may support the decision-making process and the communication to the field afterward, when discussing national strategies to improve udder health and AMU in Switzerland.

## Introduction

Bovine mastitis negatively affects milk quality (1, 2), animal welfare (3), the herd’s profitability (4), and farmers’ milking routine (5). Antimicrobial resistance (6, 7) and an increased risk of antibiotic residues in milk (8) are also associated with mastitis. Mastitis is the most common reason for applying antimicrobials to dairy cattle (9, 10). It therefore impairs the image of the dairy industry.

Bovine udder health in Switzerland is, from an international perspective, of a relative high level. Bulk milk and composite somatic cell counts are low and incidence rates of clinical mastitis are reported to be below estimates from other countries (11). The milk quality payment system in place largely explains this. Swiss farmers receive a penalty from their milk-processing company when their geometric bulk milk somatic cell count is ≥350,000 cells/ml. Some milk-processing companies have set lower penalty thresholds. On the other hand, Swiss farmers generally receive a bonus from their milk-processing companies when bulk milk somatic cell counts are <100,000 cells/ml. Despite a relatively good udder health, national annual failure costs of mastitis are estimated to be approximately 129 Million Swiss Francs for farmers, which equals to 198 Swiss Francs^1^ per average cow per year (12). Also, antimicrobial resistance of mastitis pathogens is not uncommon, especially of coagulase-negative staphylococci species for which phenotypic resistance prevalence levels up to 47% were observed (13). Finally, approximately 70% of antimicrobial usage (AMU) in dairy cows is because of intramammary purpose (14), and there is evidence that sales of intramammary antimicrobials in Switzerland are high compared with other European countries (15). Switzerland has currently a federal strategy to improve antimicrobial resistance in the human and animal populations and the environment. However, a nation-wide udder health and AMU improvement program that could improve its situation in dairy herds, especially regarding production losses and AMU, is not existing yet. Similar programs have successfully been started in many countries, including Australia (16), Canada (17), Norway (18), and the Netherlands (19).

Designing a new national animal health control program is often a highly complex and political process in which trade-offs decisions are to be made between the epidemiological and cost-effectiveness of proposed interventions on one hand, and time restrictions, financial resources, responsibilities, and stakeholders’ interests on the other hand. Issues raised are, for example, adaptation of existing legislation or payment schemes, the program’s aims and tasks (what should it do?), its implementation (who should execute it?), and its financing (who should pay for it?). Designing a new animal health program is a complex task in which various stakeholders may have different program design preferences. *A priori* investigating these preferences offers a mechanism for shared decision making (20), provides understanding of stakeholders’ opinions, and can be a starting point when discussing the program’s final design (21). Incorporating stakeholders’ preferences into the decision-making process might improve their compliance when the animal health program is implemented afterward (22). This is expected to be especially true for multifactorial animal health issues, such as bovine mastitis and AMU, where the involvement of stakeholders from the field is crucial for the success of a control program (23, 24). It is currently unclear whether a new dairy health program to improve udder health and intramammary AMU in Switzerland would be supported by the field and which components should ideally be included when an udder health and AMU improvement program is constructed.

The aim of this study was to elicit preferences of Swiss dairy farmers and veterinarians for the start and design characteristics of a new national udder health and intramammary AMU improvement program. It was also investigated whether groups of farmers and veterinarians with different start and design preferences could be identified.

## Materials and Methods

### Study Population and Sample Size Estimation

Two questionnaires were conducted in this cross-sectional study; one aiming at farmers and one at veterinarians. The sampling frame for the farmer questionnaire consisted of 19,042 dairy farmers who were producing marketed milk, had ≥11 cows, and an email address deposited at the national milk quality payment organization in May 2014 (85% of all Swiss dairy herds; personal communication by TSM Trust Ltd., Bern, Switzerland). Seasonal communal pasture holdings and herds located in the Italian-speaking Canton of Ticino were excluded. Furthermore, 1,296 dairy herds randomly selected from the same sampling frame that were requested to participate in a parallel survey were excluded from the current study to avoid farmers receiving 2 questionnaires shortly after one another. The sampling frame for the veterinarian questionnaire consisted of all 438 Swiss cattle veterinarians that were registered with the Swiss Society for Ruminant Health (Schweizerische Vereinigung für Wiederkäuergesundheit, Bern, Switzerland).

Since no prior information was available on the preference of farmers and veterinarians to start a new dairy health program, sample size calculations were estimated for the proportion with the largest variance (i.e., a proportion of 0.50). A higher level of precision was accepted for veterinarians (10%) than for farmers (5%) because lower levels resulted in sampling fractions that were deemed unachievable. The sample size needed with 95% confidence in the sample frames of 19,042 dairy herds and 438 cattle veterinarians was 385 and 79, respectively, using Winepiscope 2.0. Given an expected response rate of 30% (25, 26), 1,283 dairy farmers and 264 cattle veterinarians needed to be contacted. Using a stratified (by Swiss Canton) random sampling approach, 1,300 dairy farmers (with stratum sample sizes proportional to the cantonal dairy herd population) were eventually requested to participate. All 438 registered cattle veterinarians were contacted.

### Adaptive Choice-Based Conjoint (ACBC) Analysis

Elicitation of farmers’ and veterinarians’ preferences toward udder health and AMU improvement program characteristics was investigated using the computer-based ACBC analysis method (27) within SSI Web 8.4 (Sawtooth Software, Orem, UT, USA). In conjoint analysis, respondents make trade-off decisions between different product characteristics allowing to elicit the relative preference for each product characteristic. ACBC originates from market research and is the latest conjoint analysis technique to elicit preferences of respondents for characteristics of a product (28). Alternative conjoint techniques have been successfully applied in veterinary medicine and animal science to elicit farmers’ preferences for management strategies (29–31) and as a tool for disease prioritization (32, 33).

In conjoint analysis, products are characterized by attributes and levels (e.g., the attribute color may contain the levels red, yellow and blue for the product chair). Following conjoint analysis terminology, attributes and levels for the “product” udder health and AMU improvement program were defined as program characteristics that decision makers have to consider. Program attributes and levels were initially defined based on a literature review and the authors’ experience with national mastitis control programs. A draft list of program attributes and levels was then discussed with five experts involved in dairy cattle disease control in Switzerland, including two experts from the Federal Food Safety and Veterinary Office, two researchers from the Faculty of Veterinary Medicine, University of Bern, and one experienced practicing cattle veterinarian. The list of program attributes and levels was finalized after consulting an expert from Sawtooth Software to optimize methodological and statistical efficiency.

The two questionnaires consisted of four parts. The first part investigated the demography of respondents, their opinion on AMU in Switzerland (Tables 1 and 2), and their preference toward starting a voluntary udder health and AMU reduction program. This part included questions determining the herds’ current size and the number of treated clinical mastitis cases during the previous calendar year. The following three parts of the questionnaires concerned the ACBC interview. In the second part, respondents were offered all program attributes and levels from which they had to select their most preferred level for each attribute individually. The outcome of this part was brought forward by the software to the third part of the questionnaire. This screening section allowed the identification of program levels that were systematically avoided or preferred by respondents. Here, rather than making definite choices, alternative program designs were offered to respondents for which they had to indicate whether these were a possibility for them or not. Respondents were also asked whether such program levels were completely unacceptable or an absolute requirement for them. Program design alternatives showed after this screening section satisfied those requirements by either explicitly excluding or including program levels. Respondents were presented with a maximum of eight screening tasks, displaying four program alternatives each. These udder health and AMU improvement programs were then taken forward into the fourth and final part of the questionnaire to identify the overall best udder health and AMU improvement program. This part consisted of a traditional choice-based conjoint interview but with a restricted number of choice options to choose from since systematically avoided program levels in the screening section were excluded from this part of the questionnaire. Respondents had to choose one out of three presented program designs with a maximum of nine choice tasks (the exact number was conditional on respondents’ answers in previous sections of the ACBC interview). This facilitated discrimination of slightly different alternative program designs from the respondents’ most preferred program design (i.e., the one created in the first part of the ACBC interview). Preferred program design concepts in each choice task competed in subsequent choice tasks until the most preferred program design was identified. More detailed information on ACBC can be found in a technical paper of Sawtooth Software (28).

**Table 1:** Description of demographic and motivation characteristics of Swiss farmers.

Variable	Category	Frequency	
		*N*	%
Age (years)	0–42	161	33.7
	43–52	165	34.5
	≥53	152	31.8
Language	German	405	84.7
	French	73	15.3
Education	Certificate of competence	196	41.0
	Agricultural entrepreneur	59	12.3
	Professional degree	156	32.6
	University (of applied sciences)	14	2.9
	Other	53	11.1
Successor	Yes	153	32.0
	No	61	12.8
	Do not know yet	242	50.6
	I am the successor but have not taken over the farm yet	22	4.6
Production zone	Lowland	189	39.5
	Hilly region	96	20.1
	Mountainous region	193	40.4
Stall system	Free-stall	198	41.4
	Tie-stall	214	44.8
	Both	66	13.8
Production system	Conventional	73	15.3
	Environmental and animal friendly	345	72.2
	Organic	49	10.3
	Other	11	2.3
Dairy production is the main source of income	Yes	451	94.4
	No	27	5.7
Crop production	Yes	238	49.8
	No	240	50.2
Fruit production	Yes	91	19.0
	No	387	81.0
Poultry production	Yes	44	9.2
	No	434	90.8
Pig production	Yes	100	20.9
	No	378	79.1
Veal production	Yes	95	19.9
	No	383	80.1
Herd size (number of cows)	0–20	171	35.8
	21–30	153	32.0
	≥31	154	32.2
Incidence rate of farmer-reported treated clinical mas-titis (/100 cow-years at risk)	0–12.5	162	33.9
	12.6–23.5	150	31.4
	≥23.6	166	34.7
Do you think that anti-microbial usage is too high in Swiss dairy herds?	Yes No I do not know	164 195 119	34.3 40.8 24.9

**Table 2:** Description of demographic and motivation characteristics of Swiss ruminant veterinarians.

Variable	Category	Frequency	
		*N*	%
Gender	Male	68	69.4
	Female	30	30.6
Language	German	92	93.9
	French	6	6.1
Are you part of a joint practice?	Yes	38	38.8
	No	60	61.2
Number of vets working in practice	1	23	23.5
	2	18	18.4
	3	21	21.4
	≥4	36	36.7
Percentage of time allocated to dairy cows	0–55%	22	22.5
	55–99%	60	61.2
	100%	16	16.3
Practice is also covering companion animals?	Yes	74	75.5
	No	24	24.5
Practice is also covering horses?	Yes	75	76.5
	No	23	23.5
Practice is also covering pigs?	Yes	73	74.5
	No	25	25.5
Practice is also covering poultry?	Yes	23	23.5
	No	75	76.5
Practice is also covering exotic pets?	Yes	11	11.2
	No	87	88.8
Years working as a vet	0–10	26	26.5
	11–20	14	14.3
	21–30	34	34.7
	≥30	24	24.5
Veterinary specialization	National specialist or board certified	20	20.4
	No or other^a^	78	79.6
Proportion of antimicrobial sales being injectors	≥10%	38	38.8
	<10%	60	61.2
Do you think that antimicrobial usage is too high in Swiss dairy herds?	Yes	37	37.8
	No, sometimes, or I do not know	61	62.2

*^a^Complementary medicine, currently in education or other specializations (in other species for instance)*.

### Data Collection

Questionnaires were being conducted in German and French. Translation from German to French was conducted by a professional translator with a background in agriculture. Farmers received a personalized email explaining the purpose of the study and an individualized link to the online questionnaire in January 2015. Cattle veterinarians were contacted by email by the Swiss Society for Ruminant Health with a description of the project and a general link to the online questionnaire the same day. Reminder emails were sent after 3 and 5 weeks. To increase response rates, vouchers for an agricultural and a veterinary wholesale were provided to 110 randomly selected farmers and 50 veterinarians. Participants were also informed that they would receive a summary of the project’s results at the end of the study.

### Statistical Analysis

#### Start Preference

Preference of farmers and veterinarians to start a new voluntary udder health and AMU improvement program and their associations with potential covariates were evaluated first. Farmers’ and veterinarians’ start preference was assessed by a single question with three possible outcomes (“*The government, scientists, and the dairy industry would like to improve antimicrobial usage and udder health in Swiss dairy herds. A new voluntary program that would support farmers with this should be started. Would you be in favor of such a program?*” Answers: “Yes”, “I do not know,” and “No”). Covariates potentially associated with the start preference of farmers (Table 1) were therefore investigated using multinomial logistic regression models. Assuming a constant herd size, the herd level incidence rate of farmer-reported treated clinical mastitis was calculated as the number of treated clinical mastitis cases divided by the herd size and was expressed per 100 cow-years at risk. After a univariable screening, covariates deemed relevant (*P* < 0.25, based on the Type 3 test) were retained for further investigation. When covariate pairs had an absolute correlation >0.50, the biological more meaningful covariate was selected to avoid multicollinearity. Multivariable statistical modeling subsequently consisted of a stepwise backward elimination process until all covariates were significantly (*P* < 0.05) associated with the preference of farmers to start a new udder health and AMU improvement program or considered a confounder. Confounding was assumed to occur when effect estimates changed >25% upon exclusion of a covariate from the model. Interaction terms were not evaluated.

A similar model approach was used to associate covariates with the preference of veterinarians to start a new udder health and AMU improvement program. However, a low number of observations in the “No” category (*n* = 9) resulted in quasicomplete separation for several covariates. The outcome categories “No” and “I do not know” were therefore merged and start preference was modeled as a binary outcome variable using binary logistic regression models.

#### Part-Worth Utility Estimation

In a conjoint analysis, and thus also in an ACBC analysis, preference of respondents is quantified by part-worth utilities. Individual-level part-worth utilities represent respondent’s relative preference for each level within an attribute. Part-worth utilities are zero-centered with higher values representing more preferred attribute levels (27). Individual and mean part-worth utility values were estimated using the Hierarchical Bayes estimation procedure within Sawtooth Software (34). This estimation procedure borrows information from the entire population (prior) to determine how each respondent’s parameter estimate (posterior) differs from the upper-level population mean. It does this in an iterative manner, using a Monte Carlo Markov Chain procedure, to constantly update parameter estimates until convergence has achieved. A total of 40,000 iterations were computed, from which the first 20,000 were discarded, to obtain individual-level part-worth utility values.

The relative preference (RP*_i_*) of each attribute (*A_i_*) for each respondent was subsequently derived according to:

$${\text{RP}}_{i}=\frac{\text{Range }A_{i}}{\sum_{i=1}^{n}\text{Range }A_{i}}\times 100\%,$$

where Range *A_i_* represents the difference between the highest and lowest part-worth utility values of attribute *i*, with *n* being the number of attributes. The relative preference represents the preference each respondent has for this attribute. Preferred program-levels result in larger part-worth utility values and therefore also in a higher relative preference. Sum of the relative preference of all attributes is 100% for each respondent. Mean relative preference values were calculated for each attribute to elicit their relevance in the farmer and veterinarian population.

Goodness-of-fit of the final hierarchical Bayes models was assessed by the percent certainty and the Root Likelihood. Both indicators reflect how well a model performs in comparison to a chance model alone and a perfect model. Percent certainty is 0% for a chance model and 100% for a perfect model. Root Likelihood is 0.33 for a chance model (1 divided by the number of program alternatives per choice task, which was 3 in this study) and 1.0 for a perfect model (34).

#### Program Design Preference

The preference of farmers and veterinarians for design characteristics of a new udder health and AMU improvement program was investigated next. First, it was assessed whether farmers and veterinarians preferred certain design attributes more than others. The sum of relative preference of all attributes (eight for farmers and seven for veterinarians) for each respondent equals 100%, implying that they would be equally preferred if the relative preference of each attribute would be 12.5 or 14.3% for farmers and veterinarians, respectively. Deviation from equal preference was determined using the Wilcoxon signed rank test. The most preferred attributes were defined as having a significant mean relative preference above the equal preference threshold value. Second, differences in mean standardized part-worth utilities of program attribute levels between farmers and veterinarians were assessed using the Wilcoxon rank sum test. A Bonferroni adjustment was applied to correct for multiple comparisons.

Covariates associated with program design preferences were identified in three analytical steps. The first step was to identify a global (i.e., multivariate) association of covariates with the relative preference of the eight (seven for the veterinarians) program attributes simultaneously. The second step elicited the individual (i.e., univariate) program attributes responsible for rejection of the global null hypothesis. The third and last step was to investigate which levels within identified program attributes were associated with the covariates significant in the previous steps. Statistical modeling was performed separately for farmers and veterinarians.

Multivariate multiple regression models correct for correlation between multiple outcome variables (one for each program attribute) and multiple comparisons, thereby reducing Type 1 errors (35). Such multivariate linear regression models were used within the first step. Each potential covariate (Tables 1 and 2 plus respondents’ start preference) was tested one at a time against the relative preference of all program attributes simultaneously. The multivariate Wilks’ lambda *F* statistic was used to test the global hypothesis that all regression coefficients are zero across all program attributes for the evaluated covariate. Covariates deemed relevant (*P* < 0.25) were thereafter offered to a multivariate multiple regression in which a stepwise backward elimination process was conducted to identify all significant (*P* < 0.05) global associations between covariates and program attributes. Proportion of explained variance of the global model was derived as 1 − Wilks’ lambda, which is the multivariate counterpart of the univariate *R^2^* (35). In step 2, univariate associations of covariates with the eight (or seven) program attributes were identified for the global significant covariates using the Type 3 test commonly used for univariate linear regression models. To correct for multiple comparisons, a Bonferroni adjustment was also made in this step. Proportions of explained variance of univariate models were reported by partial eta squared [η^2^_p_ (35)]. The first two steps revealed associations between covariates and program attributes. Associations between covariates and program levels of relevant program attributes were investigated in the third and final step. Mean part-worth utility values between covariate categories were compared using the Tukey–Kramer multiple comparison test for program levels of all univariate associations identified in the previous step.

Regression modeling to associate covariates with start and program design preferences was performed using PROC LOGISTIC and PROC GLM in SAS 9.4 (Cary, NS, USA). Statistical significance was set at *P* < 0.05, except when noted otherwise. To evaluate potential non-response bias, the number of reminders (0, 1, or 2) being sent before respondents filled in the questionnaire was evaluated separately in all models evaluating the start and design preference of farmers and veterinarians.

## Results

### Demography

Of 1,300 farmers and 438 cattle veterinarians contacted initially, 478 farmers (36.8%) and 98 veterinarians (22.4%) filled out their respective questionnaires completely and were included in the statistical analysis. Seventy-three farmers were French speaking (Table 1) while only six veterinarians filled out the questionnaire in French (Table 2).

A description of farmers’ demography is presented in Table 1. Farmers’ median age was 49 (range: 26–81), and many (50.6%) did not know yet whether they had a successor or not. Most herds were located either in the lowland or mountainous regions of Switzerland. The distribution of housing systems (free-stall vs. tie-stall) was approximately equal. Dairy production was the main source of income for almost all farmers but many (82.0%) had additional agricultural production systems in place. Median farmers-reported incidence rate of clinical mastitis was 18.1 (range: 0–137.5) cases per 100 cow-year at risk.

Variables describing veterinarians’ demography are presented in Table 2. Most veterinarians filling out the questionnaire were male and working in a practice that employed multiple veterinarians. Respondents dedicated most of their time practicing dairy health but most veterinarians (96.9%) worked in practices that serviced other species too. Median years working as a ruminant veterinarian was 25 (range: 1–43).

### Preference to Start

Farmers and veterinarians were offered the following question: “*The government, scientists, and the dairy industry would like to improve antimicrobial usage and udder health in Swiss dairy herds. A new voluntary program that would support farmers with this should be started. Would you be in favor of such a program?*.” Farmers (55.4%; 95% CI: 50.1–59.8%; *n* = 265) and veterinarians (62.2%; 95% CI: 52.4–71.2%; *n* = 61) mostly agreed with this statement; 20.7% (farmers; 95% CI: 17.3–24.6%; *n* = 99) and 9.1% (veterinarians; 95% CI: 4.9–16.5%; *n* = 9) disagreed; and 23.8% (farmers; 95% CI: 20.2–27.9%; *n* = 114) and 28.6% (veterinarians; 95% CI: 17.9–50.7%; *n* = 28) did not know. These proportions were significantly different (χ^2^ = 7.16; *P* = 0.03) between the two populations with veterinarians favoring the program more.

Table 3 reports the final multivariable multinomial logistic regression model for the preference of farmers to start a new voluntary udder health and AMU improvement program. Farmers who stated that AMU in Swiss dairy herds was too high or had no opinion on this had 3.2 and 2.0 times higher odds, respectively, for preferring to start a new national voluntary program than farmers that stated that AMU was not too high. Other covariates were not statistically associated with the preference to start a new voluntary udder health and AMU improvement program.

**Table 3:** Covariates in the final multinomial logistic regression model associated with the preference (yes or I do not know vs no) of farmers to start a new udder health and antimicrobial usage (AMU) improvement program.

Covariate	Category	Preference for a new program: I do not know vs no				Preference for a program: yes vs no				*P*-value type 3 test
		OR	95% CI		Wald *P*-value	OR	95% CI		Wald *P*-value	
			Lower	Upper			Lower	Upper		
Do you think that AMU is too high in Swiss dairy herds?	Yes	1.6	0.8	3.1	0.17	3.2	1.8	5.6	<0.0001	0.0005
	I do not know	1.6	0.8	3.2	0.16	2.0	1.1	3.7	0.02	
	No	Reference				Reference				

Covariates associated with veterinarians’ preference to start a new voluntary udder health and AMU improvement program in the final multivariable logistic regression model are presented in Table 4. Ruminant veterinarians belonging to practices that also serviced poultry farms had a lower preference to start a new voluntary program than veterinarians belonging to practices not servicing poultry farms. Veterinarians who stated that ≥10% of their antimicrobial sales were attributed to the sales of intramammary antimicrobials had 3.2 times higher odds to prefer starting a new voluntary program compared to veterinarians that had <10% of their antimicrobial sales attributable to intramammary antimicrobials.

**Table 4:** Covariates in the final logistic regression model associated with the preference (yes vs I do not know and no) of veterinarians to start a new animal health improvement program.

Covariate	Category	Frequency	Preference for starting a new program (%)	OR	95% CI	
					Lower	Upper
Covering other species: poultry	Yes	23	47.8	0.3	0.1	0.9
	No	75	66.7	Reference		
Proportion of antimicrobial sales being injectors	≥10%	38	73.7	3.2	1.2	8.6
	<10%	60	55.0	Reference		

### Model Fit of ACBC

Percent certainties of the final hierarchical Bayes models estimating program design preferences of farmers and veterinarians were 46.0 and 49.5%, respectively. Root likelihood of the farmer model was 0.63 and 0.65 for the veterinarian model.

### Relative Preference of Program Attributes and Part-Worth Utilities

**Figure 1:**
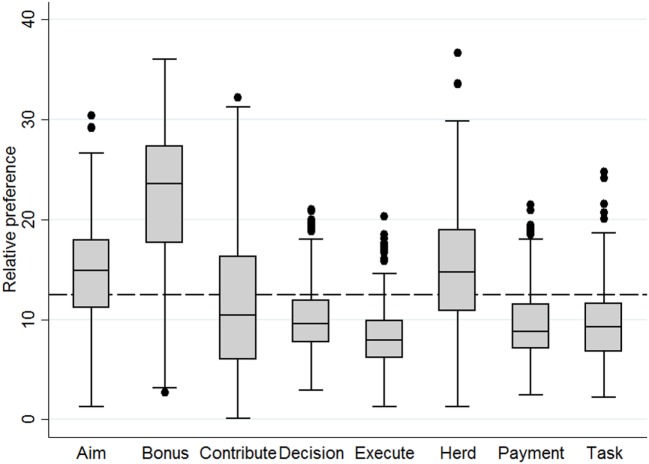
Boxplots displaying relative preference of 478 farmers for attributes of a new Swiss animal health improvement program. The dashed line at 12.5% represents an equal preference.

Relative preference of program attributes would have been 12.5% if farmers preferred them equally (the “equal preference value”). Farmers therefore preferred the program attributes “Bonus” (22.2%; 95% CI: 21.6–22.8), “Herd” (14.9%; 95% CI: 14.4–15.4), and “Aim” (14.6%; 95% CI: 14.1–15.1) more than the other five attributes (Figure 1). These three attributes had a relative preference above the equal preference value. Variation in farmers’ relative preference for attributes was large though. Within the three most preferred attributes, farmers assigned the highest part-worth utility values to levels representing a new program that did not contain a penalty system for high AMU, was voluntary for all dairy herds, and aimed to simultaneously improve udder health and reduce AMU (Table 5).

**Table 5:** Program attributes and levels evaluated in the adaptive choice-based analysis and comparison of standardized part-worth utilities for farmers and veterinarians in Switzerland.

Attribute	Description and levels	Farmers		Veterinarians		*P*-value
		Mean	SD	Mean	SD	
**Aim**	**Which aims should the program have?**					
	Improve udder health status and reduce AMU	50.9	28.0	54.7	26.0	0.18
	Reduce AMU while keeping udder health status constant	26.7	26.5	20.6	20.7	0.03
	Improve udder health status, no AMU improvement	−32.0	34.6	−42.0	30.9	0.004
	Reduce AMU, no udder health improvement	−45.5	25.3	−33.3	23.1	**<0.0001**

**Bonus**	**Should the program additionally include a bonus/malus system for AMU?**					
	No	69.6	54.0	52.3	42.4	**0.0005**
	Bonus low AMU	61.0	36.3	43.2	32.6	**<0.0001**
	Bonus low AMU and penalty high AMU	−54.2	36.2	−39.9	29.5	**<0.0001**
	Penalty high AMU	−76.5	31.7	−55.5	21.7	**<0.0001**

**Decision**	**Who should have the lead in decision making when designing the program?**					
	Dairy industry	23.4	26.2	2.0	42.0	**<0.0001**
	Breeding organizations	4.1	26.6	−29.0	22.5	**<0.0001**
	Farmers organization	0.3	24.3	−59.3	30.2	**<0.0001**
	Veterinary organization	−1.1	28.9	34.3	39.8	**<0.0001**
	University	−13.2	21.2	22.9	36.0	**<0.0001**
	Government	−13.5	27.2	29.0	38.0	**<0.0001**

**Execute**	**Who should execute the program?**					
	Dairy industry	11.9	24.8	−9.9	21.9	**<0.0001**
	Independent center of expertise	1.1	22.3	36.9	24.7	**<0.0001**
	Breeding organizations	0.8	25.3	-32.8	20.0	**<0.0001**
	Veterinary organization	−3.8	26.0	2.7	35.8	0.09
	Government	−10.0	21.1	3.2	21.0	**<0.0001**

**Herd**	**Which herds should the program target?**					
	Voluntary for all herds	46.8	45.5	12.9	39.6	**<0.0001**
	Compulsory for problem herds, voluntary for other herds	−2.1	40.4	9.6	31.7	**0.001**
	Compulsory for all herds	−44.7	35.2	−22.5	40.2	**<0.0001**

**Payment**	**Who should pay for the program?**					
	Government	21.5	29.3	5.9	27.9	**<0.0001**
	All three	11.7	23.7	23.1	18.1	**<0.0001**
	Government + dairy industry	3.6	16.4	3.5	15.9	0.85
	Government + breeding organizations	−4.9	14.0	−4.3	16.0	0.78
	Dairy industry	−6.9	28.0	7.9	21.1	**<0.0001**
	Dairy industry + breeding organizations	−8.2	17.8	−5.0	18.8	0.08
	Breeding organizations	−16.8	22.0	−31.1	19.3	**<0.0001**

**Task**	**What should be the main task for the program?**					
	Offering consulting for individual farmers	22.0	21.7	19.9	21.2	0.57
	Develop new knowledge	9.9	17.9	8.8	17.7	0.93
	Further education	5.0	20.1	8.3	18.5	0.14
	Honoring well-performing herds	−16.3	29.6	−12.9	18.7	0.02
	Mass communication	−20.6	27.3	−24.1	22.9	0.19

**Contribute**	**Mastitis in Switzerland costs on average CHF198 per cow per year. How much are you willing to contribute to the costs of the program (CHF per cow per year)?^a^**					
	CHF 0	31.7	44.3			
	CHF 1	4.3	25.7			
	CHF 2	−36.0	35.5			

*Statistically significant values (after Bonferroni adjustment; *P* < 0.0015) are presented in bold*.

*AMU, antimicrobial usage*.

*^a^This program attribute was only offered to farmers*.

**Figure 2:**
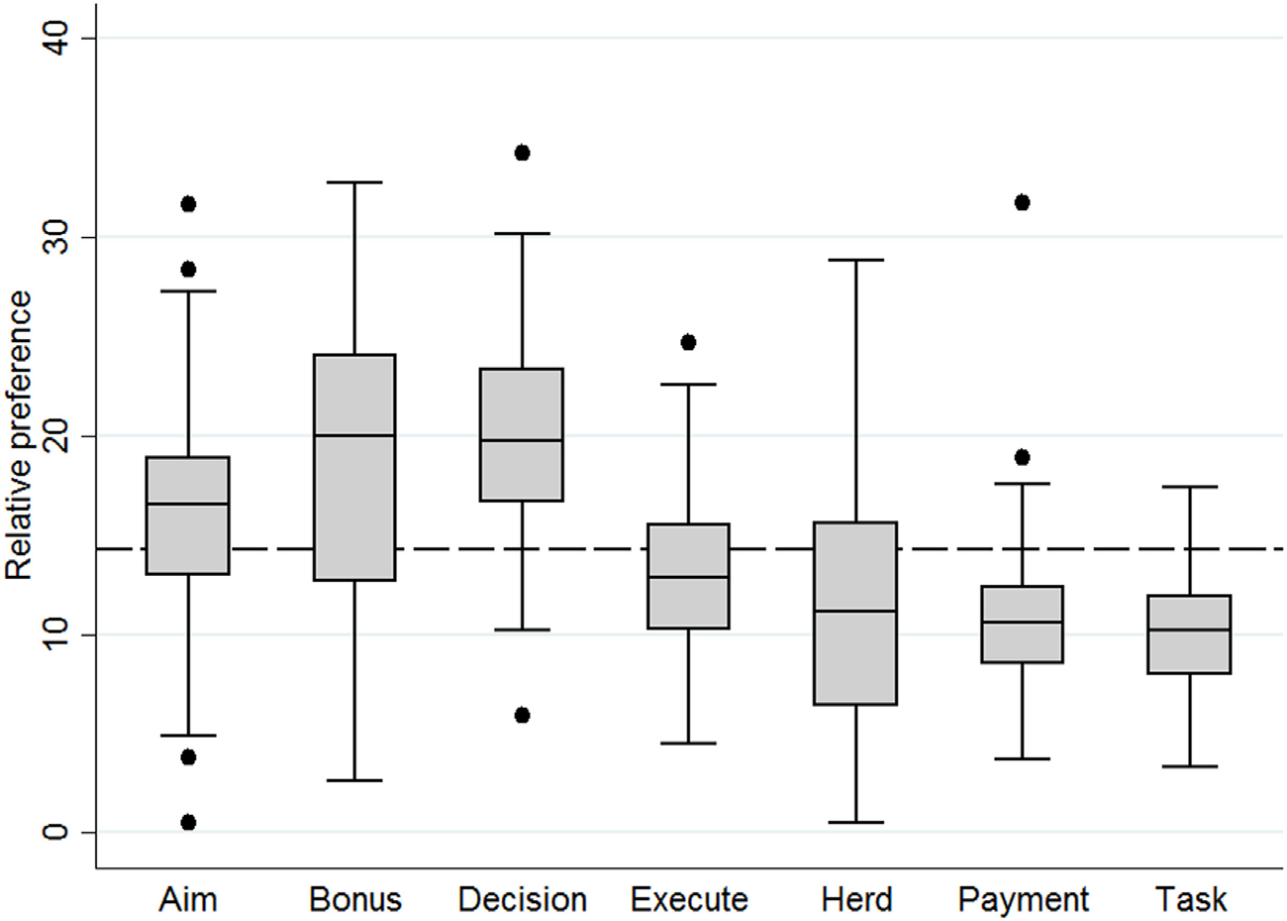
Boxplots displaying relative preference of 98 veterinarians for attributes of a new Swiss animal health improvement program. The dashed line at 14.3% represents an equal preference.

Veterinarians’ equal preference value of program attributes was 14.3%. Veterinarians preferred the program attributes “Decision” (19.8%; 95% CI: 18.7–20.9), “Bonus” (18.9%; 95% CI: 17.6–20.2), and “Aim” (16.1%; 95% CI: 15.1–17.1) more than the other four attributes (Figure 2). Like farmers’ program design preference, variation in veterinarians’ preference for program attributes was large (Figure 2). Veterinarians assigned the highest part-worth utility values to levels representing a new program that had the veterinary organization and the government taking the lead in the program design decision-making process, did not include a penalty system for high AMU, and aimed to improve udder health and reduce AMU simultaneously (Table 5).

Except for program characteristics related to the aims and tasks of the new program, farmers and veterinarians valued most program attribute levels differently (Table 5). Ranking of levels within some attributes also differed between farmers and veterinarians. This included the attribute “Decision,” which was the most preferred program attribute for veterinarians. Ranking of levels did not differ within the other three most preferred program attributes (i.e., “Aim,” “Bonus,” and “Herd”).

### Respondent Characteristics and Differences in Program Design Preferences

The final multivariate multiple regression model investigating farmers’ relative preference of program attributes is presented in Table 6. Farmers’ opinion on AMU in Swiss dairy herds was the only covariate globally associated with the relative preference of program attributes (*P* = 0.007). Further investigation of the univariate associations identified that farmers’ opinion on AMU was associated with the attributes “Bonus,” “Decision” and “Payment” (Table 6). Proportions of explained variance were low, being 0.08 for the multivariate model and a maximum of 0.04 for the univariate models.

**Table 6:** Model output of the final multivariate multiple regression model investigating farmers’ program design preferences.

	*F*	*P*	1 − Wilks’ lambda	η^2^_p_
Global model: AMU opinion^a^	2.68	0.007	0.08	
**Univariate models**				
Attribute: aim	2.95	0.05		0.01
Attribute: bonus	8.91	0.0002		0.04
Attribute: contribute	1.42	0.24		0.01
Attribute: decision	5.24	0.006		0.02
Attribute: execute	4.50	0.01		0.02
Attribute: herd	0.94	0.39		0.00
Attribute: payment	7.01	0.001		0.03
Attribute: task	1.20	0.30		0.01

*Significance in the univariate models was set at P < 0.006 to correct for multiple comparisons*.

^a^Do you think that antimicrobial usage is too high in Swiss dairy herds?

Subsequently associating farmers’ opinion on AMU with the program levels of each identified univariate program attribute revealed some significant relationships (Table 7). Farmers who had the opinion that AMU was too high in Switzerland were still not favoring the introduction of a penalty system for high AMU but their oppositions were less strong than those from farmers disagreeing with the statement that AMU was too high. Moreover, farmers agreeing with the statement that AMU was too high were slightly more in favor of the farmer organization, rather than the breeding organizations, to take the lead in decision making when designing a potential new program. Still, both groups of farmers favored the dairy industry for this. Finally, farmers who had the opinion that AMU was too high in Switzerland opposed the option that breeding organizations should pay for the program stronger than those that did not share this opinion.

**Table 7:** Mean (and SE) standardized part-worth utilities of program attributes and levels for groups of farmers with a different antimicrobial usage (AMU) opinion.

Attribute	Level	Do you think that AMU is too high in Swiss dairy herds?		
		Yes	I do not know	No
Bonus	No	51.5 (4.3)^a^	73.2 (4.6)^b^	82.7 (3.7)^b^
	Bonus low AMU	62.1 (3.1)	66.0 (3.0)	57.0 (2.5)
	Bonus low AMU and penalty high AMU	−45.0 (3.0)^a^	−56.8 (3.1)^b^	−60.3 (2.5)^b^
	Penalty high AMU	−68.7 (2.7)^a^	−82.4 (2.7)^b^	−79.4 (2.1)^b^
Decision	Dairy industry	25.0 (2.2)	22.1 (2.3)	22.8 (1.8)
	Farmers organization	3.4 (2.0)^a^	−4.2 (1.9)^b^	0.5 (1.7)^ab^
	Breeding organizations	0.6 (2.2)^a^	3.6 (2.6)^ab^	7.5 (1.7)^b^
	Veterinary organization	−0.8 (2.4)	−1.0 (2.7)	−1.5 (1.9)
	Government	−13.8 (2.3)	−9.0 (2.6)	−16.0 (1.7)
	University	−14.3 (1.7)	−11.4 (2.0)	−13.4 (1.4)
Payment	Government	20.0 (2.6)	24.2 (2.7)	21.0 (1.9)
	All three	14.7 (2.0)	11.6 (2.0)	9.1 (1.6)
	Government + dairy industry	4.5 (1.4)	3.9 (1.5)	2.7 (1.1)
	Government + breeding organizations	−5.3 (1.1)	−5.2 (1.3)	−4.3 (0.9)
	Dairy industry	−6.0 (2.3)	−8.8 (2.5)	−6.6 (2.0)
	Dairy industry + breeding organizations	−8.0 (1.6)	−9.1 (1.6)	−7.7 (1.1)
	Breeding organizations	−19.9 (1.8)^a^	−16.6 (2.0)^ab^	−14.2 (1.5)^b^

*Mean part-worth utilities within a row with various superscripts differ significantly*.

The final multivariate multiple regression model for veterinarians’ design preferences identified one borderline significant association (*P* = 0.05; 1 − Wilks’ lambda = 0.13) between the covariate describing whether veterinary practices were also servicing pig herds and veterinarians’ relative preference of program attributes. However, further investigation of the univariate associations did not reveal any significant relationship when applying a Bonferroni adjustment (data not shown). Associations of this covariate with program attribute levels were therefore not further scrutinized.

### Non-response Bias

Farmers’ preference to start a new udder health and AMU improvement program was 64.3% for respondents not receiving an email reminder (early respondents) and significantly (*P* = 0.04) decreased to 49.4% for farmers receiving two reminding emails (late respondents). Such an association was not identified for the veterinarian dataset (*P* = 0.55). The number of reminders being sent was not globally associated with farmers’ (*P* = 0.33) or veterinarians’ (P = 0.96) program design preferences either.

## Discussion

This study identified that more than half of the respondents favored starting a new voluntary national udder health and AMU improvement program. Approximately every fourth respondent was undecided and 10 (veterinarians) to 20% (farmers) disapproved of this idea. There does seem to be support from the field to initiate a new voluntary udder health and AMU improvement program when the current Swiss strategy to improve AMU and antimicrobial will be extended to dairy herds. It is unclear, however, whether these proportions are high enough to warrant an actual start of a new program. Its acceptance is likely to improve if it is accompanied with a communication campaign promoting its potential benefits (36). Moreover, the new national udder health and intramammary AMU improvement program referred to a voluntary program in which farmers would be supported with their activities improving udder health and AMU. Respondents may have been less positive if the program would have involved compulsorily activities or a more restrictive legislation. Also, only 37% of farmers answered the questionnaire, and some indication for non-response bias was found when evaluating farmers’ start preference but not for their program design preferences. The response rate was in agreement with other studies (25, 37), and demographics of farmers agreed with two previous studies investigating the same target population (25, 38) with one exception. The median farmer-reported incidence rate of clinical mastitis was higher than observed previously (11). The latter may have been a result of farmers becoming more sensitive to the topic of udder health and AMU because of a higher awareness in the farming community and society in general. They, therefore, may diagnose CM more often. Controlling bodies may have become stricter also, resulting in a potential higher reporting rate.

Response rate of veterinarians was lower at 22%, but years of experience and proportion of male respondents were in agreement with a previous survey conducted among Swiss cattle veterinarians (26). Moreover, the range in years of experience working as a dairy cattle veterinarian indicated that there was no age bias and that also older generations were reached by the online questionnaire. Indications for non-response bias were not identified in the veterinarian dataset either. It is therefore believed that the responding veterinarians represented their target population well.

Preferences of farmers and veterinarians for program design characteristics agreed moderately. Both stakeholder groups preferred a program that aimed to improve udder health and AMU simultaneously and did not include a penalty system for high AMU. These aims are in line with the strategy of the Federal Food Safety and Veterinary Office to intervene on farmers and veterinarians with a high antimicrobial consumption. Moreover, such achievements can potentially be made through the quality payment system existing in Switzerland. Farmers generally receive a bonus for their milk price when their bulk milk somatic cell count is below 100,000 cells/ml. There are no such thresholds on AMU currently. Incorporating such a threshold (after setting up a national database to register AMU on herd level) as an extra criterion for farmers to receive a financial bonus is expected to result in an improvement on AMU. Previous research has shown that farmers are more sensitive to penalties than to bonuses, as investigated for bulk milk somatic cell counts in the Netherlands (39). A penalty system might therefore be more effective in improving udder health and AMU. Nonetheless, stricter criteria for farmers to receive a bonus are also expected to result in an AMU improvement because it would take away the financial incentive to use antimicrobials to achieve a better milk price. Moreover, they are expected to be perceived less negative than receiving a penalty for high AMU (40) but this was not evaluated in the current study.

Besides a change in the milk quality payment scheme or another change in legislation, there are also other means to improve both udder health and AMU. This is evident by examples from national mastitis control programs successfully conducted elsewhere (16–19). Unfortunately, improving udder health on a voluntary basis has been proven difficult for Swiss dairy herds as identified in a recently conducted multiarm randomized field trial (24). The intervention in which farmers formed peer study group meetings to study mastitis-related topics was able to reduce AMU though while keeping the herds’ udder health status constant (24). A more realistic aim of a potential new national udder health and AMU improvement program would therefore be to reduce AMU while keeping the country’s udder health status constant. This was only the second preferred aim of both farmers and veterinarians but such achievements are feasible (24, 41). AMU and udder health are highly correlated (9, 10, 14), and any efforts to control mastitis by enhancing prevention and non-antimicrobial intervention strategies are therefore assumed to result in a decrease in AMU. Reducing AMU in dairy herds and improving its udder health status should therefore not be seen separately and be targeted simultaneously in a national control program. The program should then, however, only be evaluated on its improvement in AMU and not on its udder health improvement other than keeping it constant.

Farmers and veterinarians differed in their preferences concerning other program design characteristics. Farmers preferred a program that is voluntary. That agreed with the preference of veterinarians but this stakeholder group deemed this attribute to be of less importance. Veterinarians, on the other hand, preferred a program that had the veterinary organization and the government taking the lead in the program design decision-making process whereas the farmers preferred the dairy industry to have the lead. However, farmers generally gave a lower relative preference value to this attribute, implying that this attribute was less important to them. It is therefore believed that they would not mutually exclude each other. A voluntary program with the veterinary organization and the government having the lead in the decision-making process would still satisfy the preferences of both stakeholder groups. Other preference differences between both stakeholder groups were also identified. But those concerned again program attributes and levels that were preferred less. Not much opposition from these two stakeholder groups is therefore expected if these aspects are not fully met during the decision-making process. Moreover, incorporating these aspects in the communication of the final program design is expected to take away some of the opposition.

Farmers’ mindset toward AMU, as assessed by one single question, was the only covariate associated with farmers’ preference for starting a new udder health and AMU improvement program and for their preferred program design characteristics. None of the demographic variables explained any of the variation in start and program design preferences of farmers. Also, explained variation of the final multivariate multiple regression model was low. It can thus be hypothesized that farmers’ start and program design preferences for a new udder health and AMU improvement program may be more explained by their mindset toward udder health, AMU, or national disease control programs in general than by their demographic characteristics. Further research is needed to scrutinize this underlying sociopsychological construct. Nonetheless, some differences in start and program design characteristics between groups of farmers with various mindsets toward AMU were identified. Farmers had a stronger preference to start a new voluntary udder health and AMU reduction program when stating that AMU was too high in Swiss dairy herds or when they had no opinion on this. Those farmers, an approximate 60% of the population, therefore not only acknowledged the problem of high AMU (or were indifferent) but also supported national strategies to improve it. This included a less strong, but still existing, opposition toward the introduction of a penalty system for high AMU. Current debates in society and other strategies facilitating the recognition of high AMU could further contribute to a less strong opposition of stricter milk quality payment legislation. There were also some subtle, but significant, differences in preferences for sectoral organizations that should have a lead in decision making and that should pay for the new udder health and AMU improvement program between groups of farmers with various mindsets of toward AMU. The identification of such associations adds to the communication to the field after decision makers have discussed program alternatives.

For veterinarians, on the other hand, differences in their preference to start a new udder health and AMU improvement program according to their demographics were identified. First, the observation that veterinarians, who are earning ≥10% from their antimicrobial sales from intramammary antimicrobials, are more motivated to start a new udder health and AMU improvement program than veterinarians earning less sounds contradictory at first. An improved on-farm udder health and AMU, resulting from implementing preventive mastitis management measures as advised by a new program, are expected to result in a decreased, rather than an increased, sales of intramammary antimicrobials at the veterinary practice level. However, herd health management is not commonly applied by Swiss cattle veterinarians, and practices selling more intramammary antimicrobials are therefore assumed to have such high sales because they attempt to improve udder health in their dairy herds by treating more (e.g., subclinical) mastitis cases to lower the infectious pressure within the herd (42, 43). Increased AMU levels in Swiss dairy herds trying to improve udder health have been observed before (24). Considering the second covariate, there are only very few veterinarians servicing poultry farms in Switzerland given the small but highly organized nature of this production system. The proportion of veterinarians working at practices also servicing poultry farms (23%) therefore should be interpreted as the proportion of veterinarians working at practices servicing backyard flocks rather than specialized poultry farms. Such veterinarians may thus be less specialized in cattle health, resulting in a lower motivation to start a new national udder health and AMU improvement program. However, interpretation of both covariates identified in the final logistic regression models remains speculation, and no causal conclusions can be drawn either from this study given its cross-sectional study design. Interpretation should therefore be cautious. No global associations between covariates and veterinarians’ program design characteristics were identified, which is probably a result of the smaller sample size of this dataset (35).

Adaptive choice-based conjoint analysis was used to elicit respondents’ preferences for design characteristics of a new udder health and AMU improvement program. ACBC is a novel quantitative methodology in the field of veterinary medicine and animal science. The novel aspect lies in the adaptive nature of the interview in comparison to a standard choice-based conjoint interview (27). Respondents participating in an ACBC interview first select program characteristics that they consider most important. This consideration set is then brought forward in the remaining part of the interview in which they are jointly evaluated with alternative program designs (28). In a standard (non-adaptive) choice-based conjoint interview, a fixed number of program alternatives are offered to respondents including program attributes that may not be relevant to them (27). Results of a choice-based conjoint interview subsequently may not reflect the information that is relevant for the respondent’s situation when evaluating program design alternatives because the latter may not be close to the respondents’ ideal (28). Because ACBC interviews are more personalized than choice-based conjoint interviews, it makes them more engaging for respondents (28).

This study was limited by its cross-sectional design. Preferences of farmers and veterinarians were assessed once but may change over time resulting from discussions in society and actions implemented by governmental bodies, industry, and others. Moreover, this study assessed stakeholders’ preferences for starting a new udder health and AMU improvement program and its design. Stakeholders’ preferences of potential interventions, e.g., the creation of peer study group meetings, financial support for culling mastitic cows, more affordable diagnostics, etc., were not scrutinized. Further research is thus needed to investigate stakeholders’ preferences for the actual implementation of a program. Moreover, it was not the aim of this study to identify the perceived monetary and non-monetary benefits or disadvantages of a new udder health and AMU improvement program. Preferred design characteristics of the new udder health and AMU improvement program may thus differ from the most beneficial or practical design. Nonetheless, the results of this study facilitate discussions among decision makers. It should be noted also that this study investigated the start and design preferences of stakeholders in the Swiss context (e.g., concerning legislation and the sectoral organization of the dairy industry). Results may therefore be difficult to apply in other countries or regions, except when having a similar dairy industry. This study serves as an example on how to assess stakeholders’ preferences for new national animal health control programs.

In conclusion, most farmers and veterinarians enrolled in this survey preferred starting a new voluntary udder health and AMU improvement program in Switzerland. Particularly, they preferred a new program that aims to improve udder health and AMU simultaneously, does not contain a penalty system for high AMU, is voluntary for all dairy herds, and have the veterinary organization and the government taking the lead in the program design decision-making progress. Differences between groups of farmers and veterinarians concerning their start and program design preferences were also identified. The results of this study were not communicated with decision makers, yet they may support the decision-making process and to its communication afterward, when designing a new udder health and AMU improvement program for Switzerland.

## Ethics Statement

According to Swiss legislation, no ethical approval was required for this study since no sensitive data were collected. All participants were involved voluntarily, were informed about the research objectives of the study, and gave their consent for participation in the study. All participants were assured anonymity.

## Author Contributions

BB conceived and designed the study, collected and analyzed the data, and drafted the manuscript. All other authors provided input on the design of the study, helped interpreting study results, and critically revised the manuscript. All authors have read and approved the final manuscript.

## Conflict of Interest Statement

None of the authors of this paper have a financial or personal relationship with other people or organizations that could inappropriately influence or bias the content of the paper.

## Footnotes

^1^This equals to €182 or $199 (currency at May 1, 2017)
